# Influence of variable biochar concentration on yield-scaled nitrous oxide emissions, Wheat yield and nitrogen use efficiency

**DOI:** 10.1038/s41598-021-96309-4

**Published:** 2021-08-18

**Authors:** Khadim Dawar, Saif-ur-Rahman, Shah Fahad, Syed Sartaj Alam, Shah Alam Khan, Atif Dawar, Uzma Younis, Subhan Danish, Rahul Datta, Richard P. Dick

**Affiliations:** 1 Department of Soil and Environmental Science, the University of Agriculture Peshawar, Peshawar, Pakistan; 2 Department of Agronomy, The University of Haripur, Khyber Pakhtunkhwa, Pakistan; 3 Department of Plant Pathology, The University of Agriculture Peshawar, Peshawar, Pakistan; 4 Depertment of Plant Protection, The University of Agriculture Peshawar, Peshawar, Pakistan; 5 Department of Botany, University of Central Punjab, Lahore, Punjab Pakistan; 6 Department of Soil Science, Faculty of Agricultural Sciences and Technology, Bahauddin Zakariya University Multan, Punjab, Pakistan; 7 Department of Geology and Pedology, Faculty of Forestry and Wood Technology, Mendel University in Brno, Zemedelska1, 61300 Brno, Czech Republic; 8 School of Environment and Natural Resources, Ohio State University, Columbus, Ohio USA

**Keywords:** Plant sciences, Environmental sciences

## Abstract

An important source of the destructive greenhouse gas, nitrous oxide (N_2_O) comes from the use of ammonium based nitrogen (N) fertilizers that release N_2_O in the incomplete conversion (nitrification) of NH_4_^+^ to NO_3_ˉ^1^. Biochar has been shown to decrease nitrification rates and N_2_O emission. However, there is little information from semi-arid environments such as in Pakistan where conditions favor N_2_O emissions. Therefore, the object was to conduct field experiment to determine the impact of biochar rates in the presence or absence of urea amended soils on yield-scaled N_2_O emissions, and wheat yield and N use efficiency (NUE). The experiment on wheat (*Triticum aestivum* L.), had a randomized complete block design with four replications and the treatments: control, sole urea (150 kg N ha^−1^), 5 Mg biochar ha^−1^ (B5), 10 Mg biochar ha^−1^ (B10), urea + B5 or urea + B10. In urea amended soils with B5 or B10 treatments, biochar reduced total N_2_O emissions by 27 and 35%, respectively, over the sole urea treatment. Urea + B5 or + B10 treatments had 34 and 46% lower levels, respectively, of yield scaled N_2_O over the sole urea treatment. The B5 and B10 treatments had 24–38%, 9–13%, 12–27% and 35–43%, respectively greater wheat above-ground biomass, grain yield, total N uptake, and NUE, over sole urea. The biochar treatments increased the retention of NH_4_^+^ which likely was an important mechanism for reducing N_2_O by limiting nitrification. These results indicate that amending soils with biochar has potential to mitigate N_2_O emissions in a semi-arid and at the same time increase wheat productivity.

## Introduction

The world is facing unparalleled challenges to provide food security while conserving soil and water resources for food production; United Nations 2017. To address this, sustainable cropping systems are needed that, besides promoting resource conservation, also increase food, fuel, and fiber productivity. In Pakistan, these challenges are exacerbated by climate change and rapidly growing rural populations causing land scarcity. This fits into a worldwide issue to grow more food for an ever-increasing population.

To address the challenge of increasing food production, a key agricultural input for maximizing crop production is the use of inorganic fertilizers, particularly nitrogen (N). Lin et al.^1^ estimated a need to apply high rates of N fertilizers (290–349 kg N ha^−1^ yr^−1^) to maximize crop production. However, these high N rates typically exceed crop N demand, resulting in low NUE, and high ammonia (NH_3_), and N_2_O^2,3^ losses that have negative environmental impacts^4,5^.

Nitrous oxide is important because it is a persistent greenhouse gas with about 298 times more global warming potentials than CO_2_ that destroys ozone (O_3_) and causes up to 7% of the greenhouse effect from anthropogenic activities^6^. It is increasing by 0.26% per anuum^−1^^7,8^, with agricultural soils being the highest source (65–70%) (4.1 Tg N year^−1^)^9^ of the total N_2_O emissions from terrestrial ecosystem^10,11^. This is mainly due to the extensive use of N fertilizers, particularly when applied at high rates in soils that have low soil pH, low carbon (C) availability, and high moisture content (anaerobic condition)^12,13^. Improved soil management systems are needed that would reduce or eliminate N_2_O production from agricultural soils.

One possible approach to control N_2_O emissions is the application of biochar which is produced from various organic materials (e.g. plant residue, manure) by pyrolysis under anaerobic conditions. When added to soils, biochar has been shown to stabilize and store inorganic nutrients resulting in greater nutrient uptake efficiency in crops^14–19^. Furthermore, it decomposes slowly that results in beneficial properties being sustained for long periods^20^. An important favorable characteristic is that it has a large surface area enabling greater adsorption of anions and cations^21–24^, and absorption of the greenhouse gases, CO_2_ and N_2_O^25^. There is also evidence that biochar increases the growth and activity of bacteria and fungi, which involved in the mineralization of N^26^.

Of particular interest is that biochar could affect denitrifying microorganisms, especially those that have the nosZ gene^27^. This gene codes for the key enzyme, nitrous oxide reductase, that mitigates N_2_O emissions by catalyzing N_2_O reduction to benign N_2_ gas. Biochar has been shown to be very effective at trapping greenhouse gas emissions from agricultural soils^28^. Estimates by Cayuela et al.^25^ on laboratory and field studies using meta-analysis indicated potential reductions of N_2_O emissions due to biochar soil was 54 and 28%, respectively.

However, there has been very little research on the effect of biochar in controlling N_2_O losses from soils treated with the widely used N fertilizer, urea, under the hot climatic conditions as found in Pakistan. Therefore, the objectives of this study were to investigate the effect of biochar on N_2_O emissions, crop productivity, and N use efficiency.

## Results

### Soil moisture and temperature

**Figure 1:**
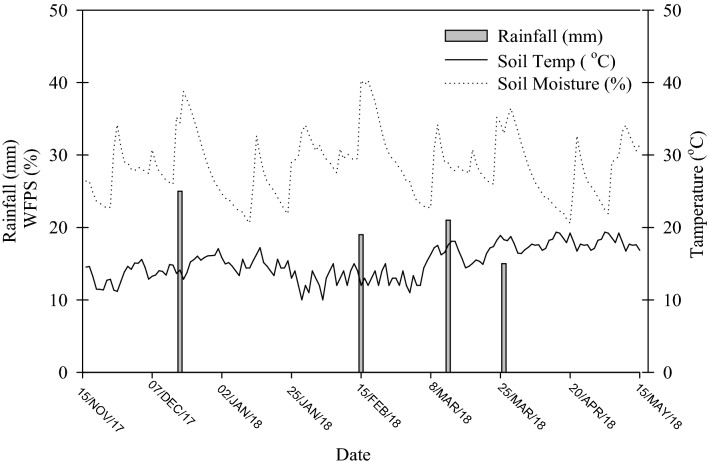
Soil temperature (0–10 cm depth) and moisture (water-filled pore space; WFPS) (0–10 cm depth) and monthly average precipitation during the experimental period.

Within the wheat growing season mean temperature at 10 cm depth soil was 13.0 °C ranging from 11.5 to 19.5 °C, and total precipitation was 101 mm (Fig. 1). Mean soil WFPS at 10 cm depth throughout all treatments was 41%, with maximum of 49% occurring in irrigated soil just after first fertilizer application.

### Inorganic N dynamics

**Figure 2:**
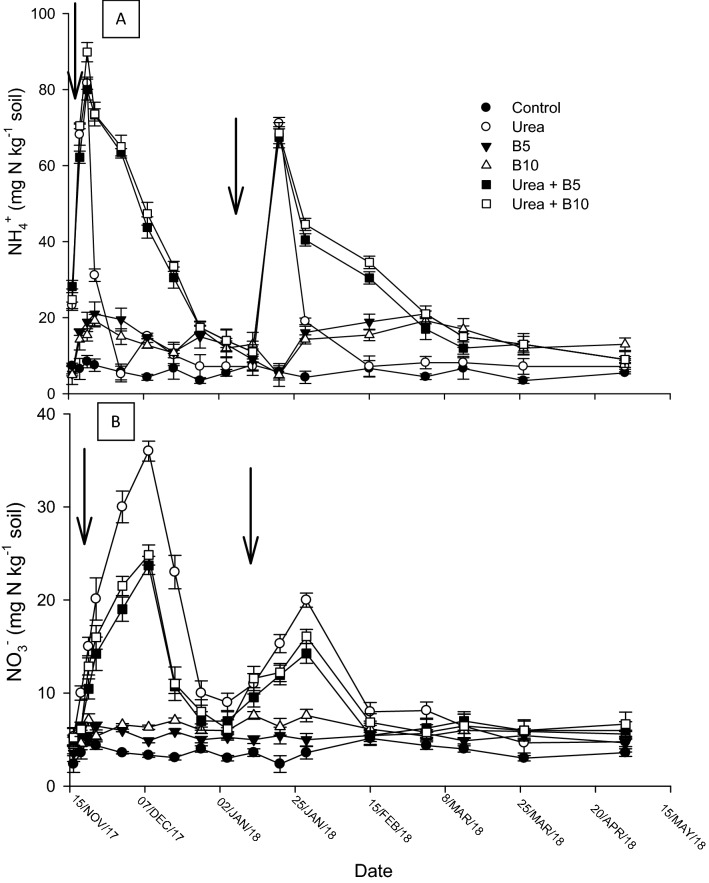
Soil NH_4_^+^ and NO_3_^-^ (0–10 cm depth) in soils amended with sole urea or urea + biochar treatments. Solid arrows show time of N fertilization.

One day after urea application, the concentration of NH_4_^+^–N significantly (*P* < 0.05) increased (5–25 mg N kg^−1^ soil) over the control treatment. Soil NH_4_^+^ concentration in the urea treatment reached a maximum on day three and decreased after that. Soil NH_4_^+^ concentrations remained significantly (*P* < 0.05) higher up to 28 days in the urea-biochar treatments. The sampling at pre-plant-urea application had higher soil NH_4_^+^ concentrations than the second urea application. Average soil NO_3_^-^ concentrations ranged from 5.9 to 36.8 mg N kg^−1^ Fig. 2B. Across all treatments NO_3_^-^ concentration was significantly (*P* < 0.05) higher at 14 days after fertilizer application, after which gradually returned to background levels. After basal fertilization, the highest soil NO_3_^-^ concentration was recorded in the sole urea treatment.

### N_2_O emissions

**Figure 3:**
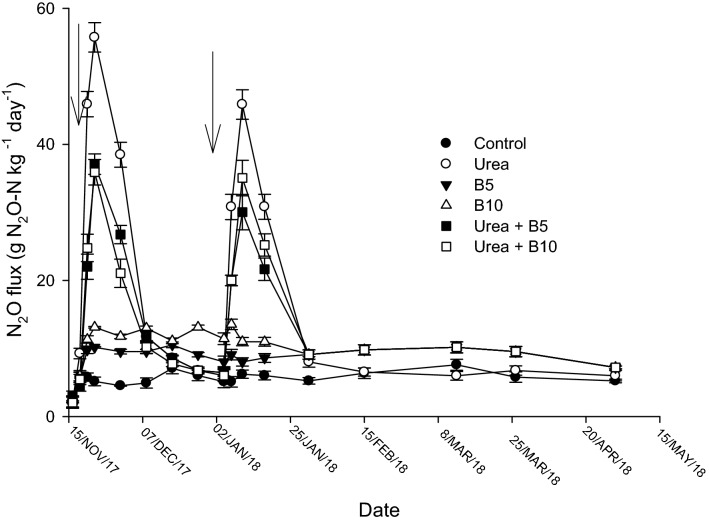
Fluxes of N_2_O. in soils amended with sole urea or urea + biochar treatments. Solid arrows show time of N fertilization.

Nitrous oxide (N_2_O) emissions had temporal variations that were similar across all treatments over the course of the experiment (Fig. 3). The N_2_O fluxes were generally two distinct peaks that occurred after the urea application. Cumulative N_2_O emissions in the urea-amended treatments varied from 0.46 to 0.67 kg N ha^−1^ which were significantly (*P* < 0.05) higher than the control (Table 1). Over the 150-day experimental period, the highest total N_2_O emission occurred in the sole urea treatment. The 5 or 10 Mg biochar ha^−1^ rates, when combined with urea had total N_2_O emission of 0.50 kg ha^−1^ and 0.46 kg ha^−1^, respectively which, reduced N_2_O emission by 27% and 34%, respectively, over the sole urea treatment. However, adding biochar by itself increased N_2_O emissions by > four times the control. The yield-scaled N_2_O emission, based on the cumulative N_2_O emission and the above ground N uptake), ranged from 7.4 (± 0.7) to 4.0 (± 0.5) g N_2_O–N kg^−1^ over the entire experimental period (Table 1). Urea applied with biochar, either 5 or 10 Mg ha^−1^ significantly (*P* < 0.05) decreased the yield-scaled N_2_O emission by 34 to 46% relative to sole urea over the entire experimental period.

**Table 1:** Total N_2_O-N release, N emitted as N_2_O, % difference comparative to urea and yield scaled N_2_O emissions in soils amended with sole urea or urea + biochar.

Treatment	N_2_O emission (kg N ha^−1^)	N emitted as N_2_O of the applied N (%)	% difference over urea	Yield-scaled N_2_O emission	% difference over urea
Control	0.05 ± 0.02e		–		
Urea	0.67 ± 0.12a	0.41	–	7.4 ± 0.7^a^	
B 5	0.21 ± 0.08d	0.10			
B 10	0.22 ± 0.09d	0.11			
Urea + B 5	0.50 ± 0.13b	0.30	− 27	4.9 ± 0.4^b^	34
Urea + B 10	0.46 ± 0.12c	0.27	− 35	4.0 ± 0.5^bc^	46

Within columns, means followed by the same letter are not significantly different at *P* < 0.05.

### Wheat productivity

Grain yield of wheat was significantly (*P* < 0.05) higher when urea was applied with biochar than the sole urea treatment (Table 2). Urea with biochar at the 5 and 10 Mg ha^−1^ rates had grain yields of 4151and 4327 kg ha^−1^, respectively which were significantly (*P* < 0.05) greater than the 3827 kg ha^−1^ produced by sole urea.. Similarly, the biochar-urea treatment had a significant (*P* < 0.05) effect on wheat straw yield (Table 2). Maximum straw yields of 5704 kg ha^−1^ and 6652 kg ha^−1^ were recorded, when urea was applied with biochar, 5 and 10 Mg ha^−1^, respectively, which was 39% and 62% (respectively) greater the sole urea treatment.

**Table 2:** Total aboveground biomass, and grain and straw yield (kg ha^−1^) in soils amended with sole urea or urea + biochar treatments.

Treatments	Biological yield	% increase over urea	Grain yield	% increase over urea	Straw yield	% increase over urea
Control	5604 ± 211a		2683 ± 9a		2921 ± 43a	
Urea	7930 ± 228b		3827 ± 59b		4103 ± 65b	
B 5	7117 ± 209c		3162 ± 52c		3955 ± 55c	
B 10	7731 ± 176d		3328 ± 59d		4403 ± 62d	
Urea + B 5	9855 ± 216e	24	4151 ± 69b	9	5704 ± 79b	39
Urea + B 10	10,978 ± 226a	38	4327 ± 59b	13	6652 ± 59b	62

Values within a column followed by the same letter are not-significantly different at *P* < 0.05.

Urea applied with biochar had a significant effect on plant height, 1000 grain weight, and number of grain per spike^-^of wheat compared to the urea-alone treatment (Table 3). The highest plant height was recorded for urea + biochar 10 Mg ha^−1^ (73.3 cm), followed by urea + biochar 5 Mg ha^−1^ (65.9 cm), which was 10% and 5%, respectively greater than the sole urea treatment. Similar positive biochar treatment effects were found for grain weight and number of grains spike^−1^ (Table 3).

**Table 3:** Plant height (cm), number of grains spike^-1^, 1000 grain weight (g) in soils amended with sole urea or urea + biochar treatments.

Treatments	Plant height (cm)	% increase over urea	No. of grains spike^−1^	% increase over urea	1000 grain weight (g)	% increase over urea
Control	28.7 ± 5a		19.3 ± 4a		49 ± 9a	
Urea	59.6 ± 9b		30.4 ± 5b		90 ± 11b	
B 5	33.5 ± 7c		24.6 ± 7c		58 ± 8c	
B 10	36.3 ± 5c		28.7 ± 6c		62 ± 5bc	
Urea + B 5	65.9 ± 8d	5	35.1 ± 5d	18	101 ± 15b	7
Urea + B 10	72.3 ± 6e	10	41.7 ± 7e	25	114 ± 11e	9

Values within a column followed by the same letter are not-significantly different at *P* < 0.05.

### N uptake by plant with N use efficiency

Urea applied with biochar had a significant effect on N content of grain, straw and total biomass of wheat compared to the urea-alone treatment (Table 4). All urea fertilizer treatments increased the total N uptake in above-ground biomass compared with the control treatment. The maximum total N uptake of 114 kg ha^−1^ was the urea + biochar at 10 Mg ha^−1^ followed by urea with biochar 5t. Biochar significantly (*P* < 0.05) increased total N uptake from 12 to 27% of wheat over the sole urea treatment (Table 4). Nitrogen use efficiency of urea-N was 35 and 43 for B10 and B5, respectively compared to 27 for the sole urea.

**Table 4:** Grains, straw, and total N uptake (kg ha^-1^), and NUE (kg N uptake kg^−1^ of applied N) in soils amended with sole urea or urea + biochar treatments.

Treatments	Total N in grains	Total N in straw	Total N uptake	Percent increase over urea	NUE (%)
Control	28.7 ± 6a	19.3 ± 2a	49 ± 7b		
Urea	59.6 ± 9b	30.4 ± 5b	90 ± 9b		27
B 5	33.5 ± 5c	24.6 ± 4b	58 ± 6b		6
B 10	36.3 ± 7b	28.7 ± 5b	62 ± 5b		9
Urea + B 5	65.9 ± 8b	35.1 ± 6b	101 ± 16b	12	35
Urea + B 10	72.3 ± 5b	41.7 ± 7b	114 ± 8b	27	43

Values within a column followed by the same letter are not-significantly different at *P* < 0.05.

## Discussion

### N dynamics

Urea was rapidly hydrolyzed to NH_4_^+^ within 2–3 days of urea application at the experimental site, which resulted in greater soil NH_4_^+^ levels in urea amended soils, with or without biochar amendments (Fig. 2A). This is likely due to optimal soil moisture levels at the time of the experiment (Fig. 1). After day 7, soils that received urea + biochar treatment had significantly more NH_4_^+^ than sole urea which continued until day 28. The higher retention of NH_4_^+^ in the presence of biochar can be attributed to the greater surface area and cation exchange capacity of biochar amended soils for adsorption of NH_4_^+^. Also, there is evidence that biochar directly inhibits nitrification by immobilization and adsorption of inorganic N by biochar and to the genus *Rhodococcus* of *nirK-type* denitrifiers^29,30^. Ding et al.^31^ assessed that biochar could absorb NH_4_^+^–N by its exchange capacity resulted in minimization of NH_4_^+^–N downward movement in soil deeper layers.

The application of urea + biochar significantly enhanced yield by increasing total N uptake and NUE by the plant compared to the sole urea treatment (Tables 2 and 4). The finding of previous studies also showed that the use of biochar with fertilizer increased the plant yield^32^. Similarly, another study also suggests the role of biochar + fertilizer in increasing micronutrient availability, soil pH with more water-holding capacity, and less concentration of exchangeable Al^33^. This increase may be due to an increase in the concentration of mineral N →NH_4_^+^ form than in NO_3_^-^ just after few days of urea + biochar application (Fig. 2), thereby increasing the N uptake and crop yields^34^. Ammonium retention in soil due to biochar application provides environmental benefits by reducing NH3 and N2O emission and NO3- leaching 45,46, but also offers agronomic and economic benefits by increasing NUE especially in N deficient soils^35^.

### Wheat productivity

Wheat grain and straw production was significantly increased by the addition of biochar with or without the addition of urea. That this response occurred without an external N input with urea, suggests that biochar is having beneficial effects for crop growth beyond N use efficiency. One factor could be that biochar improves soil structure and fertility^31^; and in turn an improved rooting environment and possibly many other benefits that are associated with improved physical structure (e.g. improved microbial habitat, water storage, gas exchange) could account for this plant growth response due to biochar additions to soils.

The improved yield, when urea was added with biohar, could be related to the improved retention of NH_4_^+^ which is supported by the soils data. Biochar increased retention of NH_4_^+^ that by itself could improve N uptake by wheat storing this form of N and not being converted to NO_3_^−^ that is susceptible to leaching. Furthermore, plants use less energy when NH_4_^+^ is taken up instead of NO_3_^−^ because conversion of NH_4_^+^ into amides, amines and amino acids is more efficient and uses less energy than NO_3_ in plants^35,36^.

### N_2_O emissions

As expected, experimental plots fertilized with urea had higher N_2_O fluxes than the control. Emissions of N_2_O reached their peak 2–3 days after fertilizer application (Fig. 3), when there was high soil moisture followed by a decline which corresponded to a reduction rainfall (Fig. 1). This decreasing soil moisture has been shown to decrease diffusion and hydrolysis of urea that results in lower conversion to N_2_O instead of NH_4_^+^^37^. Total N_2_O emission was lowest at 0.05 kg N_2_O–N ha^−1^ in the control and highest at 0.67 kg N_2_O–N ha^−1^ in the sole urea treatment.

Urea + biochar significantly reduced total N_2_O emissions by 27 to 35% over the sole urea treatment (Table 1). A likely mechanisms for this response is that soil amended with urea + biochar retained greater NH_4_^+^ over sole urea amended soil, that in turn reduced N_2_O emissions inhibiting NH_4_^+^ from being available for nitrification. Thus avoiding this reaction that produces N_2_O under aerobic or anaerobic conditions^29,38^. These results correspond to Zhang et al.^39^, who showed that biochar (10–40 Mg ha^−1^) significantly decreased the N_2_O–N emissions (31–58%). Lastly biochar amended soils increased soil pH, which enhances the enzyme (N_2_O-reductase) that converts N_2_O to N_2_^40^ and thus could be a further mechanism for biochar to reduce N_2_O losses from soils.

The yield-scaled N_2_O emission as the amount of N emitted as N_2_O divided by the total N uptake by the aboveground biomas in the present was within 4 to 7.4 g N_2_O–N kg^−1^ across all treatments (Table 1). This is similar with other reported estimates of 5 to 15 g N_2_O–N kg^−1^^41–43^. Urea + biochar minimized yield-scaled N_2_O emission significantly by 34 to 46% over sole urea (Table 1). These results agree with previous studies^44^ that compared the effect of biochar and a nitrification inhibitor on yield-scaled N_2_O emissions in a vegetable field over 2 years in southeastern China. Similarly, there was a negative correlation between yield-scaled N_2_O emission and NUE, indicating that the N that would be lost as N_2_O is being taken up by the crop—thus having positive environmental and agronomic effects.

The biochar treatments in the absence of urea, significantly increased N_2_O emissions. However, it seems that there was a basal level of production that did not increase significantly with B10 over B5 (Table 1). This is note worthy, in that although biochar reduced N_2_O emissions of urea-N, based on the above observation some portion of this emission was coming from the biochar itself. This is an important finding as it means that future research should determine which type of biochar substrate and/or production method (e.g. temperature and other conditions) minimizes or eliminates N_2_O emissions.

Nitrous oxide is an important greenhouse gas because it reduces the ozone layer and has 300 times the heat-trapping capability of carbon dioxide^45^. Crop production systems that use inorganic N fertilizers that contain or produce (e.g. urea) NH_4_^+^ is a major source of N_2_O emissions. This is because NH_4_^+^ is the substrate for nitrification during which N_2_O can be produced^46^. Hence, use of biochar holds potential as an important agronomic management tool to reduce N_2_O emissions from cropped soils and improve NUE.

## Conclusions

Semi-arid regions of Pakistan with its hot weather conditions are very favourable for the production of the greenhouse gas, N_2_O (gas) when urea fertilizer is added to soils. The results of this study showed that amending soils with biochar significantly reduced N_2_O losses and yield-scaled N_2_O emissions. Similarly, urea + biochar significantly improved wheat grain and straw productivity. and enhanced plant N uptake over the sole urea treatment. In conclusion, biochar was shown to significantly reduce N_2_O losses from soils amended with urea-N fertilizer while improving N use efficiency and wheat and productivity. The results support further long = term experiments in Pakistan on biochar as management tool to determine its effects on specific soil properties and mechanisms for controlling N_2_O emissions and promoting plant growth. Further research is needed to determine the importance of biochar type in controlling N_2_O emmistions.

## Methodology

### Experimental site

Current sturdy was done in the research area (34.1°’21″ N, 71°28′5′ E) of University of Agriculture, Peshawar, Pakistan from Nov. 2017 to May 2018. The characteristics of experimental soil are provided in Table 5. Climate of area was semi arid with 380–400 mm year^−1^ rainfall. The site is 350 m above sea level with average annual temperature of 23 °C. The site has been under an irrigated maize-wheat cropping rotation for nearly 15 years. The treatments include urea at a rate of 300 or 150 kg N ha^−1^ applied to *Zea mays* L. and *Triticum aestivum* L., respectively.

**Table 5:** Soil physiochemical characteristics.

Attributes	Units	Value
Sand	%	28.2
Clay		8.1
Silt		63.7
Texture	–	Silt loam
pH*s*	–	7.75
CEC	μS cm^−1^	150
Organic matter	%	0.84
Total nitrogen		0.07
Extractable P	μg g^−1^	3.67

### Biochar production and characteristics

Biochar was produced from pyrolyzing the acacia tree prunings collected as biowaste from the farm. The acacia tree prunings were air-dried before pyrolyzing at 450 °C for 90 min and then using a muffle furnace at 550 °C under limited oxygen supply. Biochar was passed through a 5.0-mm mesh. pH and electrical conductivity (EC) of biochar were measured on 1:10 ratio (w/v) suspension using digital pH (InoLab, WTW Series, Germany) and EC (Jenway, UK) meters. Total organic C and total N contents of biochar was measured on a Vario Micro CHNS-O Analyzer (Elementar Analysensysteme GmbH, Hanau, Germany). To determine total P and K contents, biochar samples were digested in hydrogen peroxide and sulfuric acid solutions. Total P contents in supernatants were measured on a UV–visible spectrophotometer (Shimadzu, Tokyo, Japan), whereas K contents were measured on a flame photometer with a 0.2-ppm detection limit (Jenway, Cole-Parmer, UK). The digested samples were also used to determine Na^+^, Ca^2+^, and Mg^2+^ on an inductively coupled plasma-atomic emission spectroscopy (ICP-AES, Agilent, USA). The pH and EC of biochar were 7.35 and 1.25 dS m^−1^, respectively. Analyses showed that the total C, N, P, and K contents of biochar were 568, 2.40, 6.91, and 5.19 g kg^−1^ whereas Na^+^, Ca^2+^, and Mg^2+^ contents were 3.80, 12.3, and 7.21 g kg^−1^, respectively.

### Experimental design and management

The experiment had a randomized complete block design with four replications and the following treatments: (1) control (zero N), (2) urea (150 kg N ha^−1^, U), (3) biochar 5 Mg ha^−1^ (B5), (4) biochar 10 Mg ha^−1^ (B10), (5) U + B5 and (6) U + B10. The plot size was 5 × 3 m^2^ containing 10 rows (5 m long and 30 cm spacing) where each plot had an irrigation entrance and exit for drainage. A 0.5 m^2^ area was allocated for soil sampling. The soil was cultivated by tine ploughing up to a depth of 0.30 m, followed by two y 2-cultivations across the field and planking was done in all plots to break the clods. Before sowing, surface irrigation of 100 mm was applied and the final seedbed was prepared when soil moisture reached field capacity after six days of irrigation (i.e., 50% WFPS). At the same time, a basal application of phosphorus (P) at 90 kg P_2_O_5_ ha^−1^ in the form of single superphosphate and potassium (K) at 60 kg K_2_O ha^−1^ in the form of potassium sulphate (K_2_SO_4_) were applied and incorporated into the soil. The biochar treatment, was incorporated by ploughing to a depth of 20 cm. The wheat variety Pirsabak-2013 was planted at a seed rate of 120 kg ha^−1^ on 15^th^ November 2017 using a mounted planter equipped with row cleaner wheels. Urea was applied as a split application of 75 kg N ha^−1^ at planting and 75 kg N ha^−1^ at tillering stage.

### Nitrous oxide gas sampling, analysis and flux calculation

Soil N_2_O gas fluxes were measured from November 2017 to May 2018 using a closed chamber method as described by Saggar et al.^47^ Gas samples were collected on 0, 1, 3, 5, 7, 10 and 15 days after each urea application followed by once a week sampling until wheat maturity. Gas sampling was consistently performed between 8:30 and 10:00 h, to minimize diurnal variation and better represent the mean daily fluxes. After each fertilizer application, N_2_O fluxes were initially taken three times during the 1st week and later, weekly or twice monthly. A PVC chamber (15 cm long × 30 cm wide with 450 cm^2^ area) was inserted into the soil about 5 cm deep between wheat rows on the perimeter of each field plot. The chamber was composed of two separate compartments joined together with an airtight rubber septa for measuring to daily N_2_O accumulation. The chamber had two ports; a small silicon sealed vent for sampling and a second port for measuring soil temperature in the chamber.

During sampling, 3 gas samples were collected at 0, 30 and 60 min time from each chamber via 50 mL polypropylene syringes equipped with 3-way stopcocks. Ambient air sample was collected precisely after closing the chambers (time 0). It was utilized as a reference for determining N_2_O gas fluxes. During each sampling, temperature of chamber was recorded by using mercury thermometer. Gas samples were instantly shifted to pre-evacuated 20 mL glass vials (molded PTFE/black butyl septum, Agilent Technologies, USA). Gas samples were examined in an Agilent 7890A gas chromatograph (Agilent Technologies, Santa Clara, CA, USA) supplied with an electron capture and detector and a mechanized connected to a flame ionization detector headspace auto-sampler Agilent 7697A (Agilent Technologies, Santa Clara, CA, USA).

The cumulative N_2_O emissions were calculated by summing all daily fluxes for the experimental period and assuming that the daily fluxes changed linearly when no daily data were available. The N_2_O flux (μg N_2_O m^-2^ h^−1^) was calculated according to the equation as per Dawar et al.^41,]^^43^ as follows:

1 $${\text{Flux}} = \frac{{{\text{dGas}}}}{{{\text{dt}}}} \times \frac{{{\text{V}}_{{{\text{chamber}}}} \times {\text{ p }} \times 100 \times {\text{ MW}}}}{{{\text{R}}*{\text{T}}}} \times 10^{3} \times \frac{1}{{\text{A}}}$$

where: *dGas* is change in ppb concentration over time; 10^3^ is a unit conversion factor; *V*_*chamber*_ is chamber volume; *p* is atmospheric pressure in Pa; *MW* is the molecular weight of N_2_O-N; *R* is the gas constant 8.314 J mol^−1^ K^−1^; *T* is the temperature in Kelvin; *A* is a chamber basal area o.

The yield-scaled N_2_O emissions were calculated as the amount of N emitted as N_2_O divided by the total N uptake by the aboveground biomass^48^. The emission factor (EF) of N_2_O was determined following the IPCC (2006) Tier I methodology as follows:

2 $${\text{N}_{2}\text{O}}\,\,{\mathrm{EF}}(\%)=\frac{{\text{N}}_{2}\text{O}-\text{N treatment}-{\text{N}}_{2}\text{O}-\text{N control}}{\text{N input}}\times 100$$

where: N_2_O–N treatment is N_2_O emissions (kg N_2_O–N ha^−1^) in N treatment plots; N_2_O-N control is N_2_O emissions (kgN_2_O–N ha^−1^) in control plots, N input is the amount of N (kg N ha^−1^) applied to N treatment plots.

### Wheat measurements

The wheat crop from main plots (5 × 3 m^2^ containing 10 rows (5 m long and 30 cm spacing)) was harvested at physiological maturity in May 2018 and data recorded on above ground biomass (shoots and leaves) and grain yield. Whole plants of the central four rows were harvested and then thrashed to obtain grain yield. Five randomly chosen plant sub-samples (ca. 1000 g fresh weight) from each sub-plot were transferred to sealed plastic bags and transported on ice to ensure no water losses plant water loss. Total fresh plant biomass weight was immediately recordedand then dried at 65 °C for seven days followed by measurement of the dry weight. Grain moisture was determined with a moisture meter to calculate dry grain yield. Straw (leaves plus stems) yield was calculated by subtracting grain yield from the total biomass yield of wheat. N content was determined separately for above-ground biomass (shoot and leaves) and grain by first grinding to pass a 100 mesh sieve, followed by total N analysis with the Kjeldahl method.

Nitrogen use efficiency was calculated as follows:

3 $$\text{NUE }(\text{\%})=\frac{\text{N uptake by fertilized crop }-\text{N uptake by unfertilized crop}}{\text{Amount of fertilizer applied}}\times 100$$

Thousand grains weight was recorded by weighing 1000 randomly selected grains. Plant height was recorded and averaged on five randomly selected plants at physiological maturity. Spike length was measured on five spikes randomly selected from each plot from the base of the rachis to the tip of the uppermost spikelet. Five randomly selected spikes from each plot were thrashed individually to determine the number of grains per spike and then averaged.

### Plant and soil analysis

Before starting the experiment, four composite soil samples (0–10 cm depth) were taken using a soil core from the experimental site and passed through a 2-mm sieve.

Soil samples were randomly collected from the experimental site before treatments were imposed. Analysis for basic soil properties (Table 5) of pH and EC were measured on 1:5 ratio (w/v, basis) in water by using a pre-calibrated pH (InoLab, WTW Series, Germany) and EC meter (Jenway, UK) with the protocol described by Page et al.^49^ and Rhoades^50^. Soil organic matter was determined by the method described by Nelson and Sommers^51^. One g of soil was mixed with 10 mL of 0.5 N K_2_Cr_2_O_7_ and 20 mL of concentrated H_2_SO_4_. The mixture was then left for 30 min and allowed to react completely. This step was followed by the addition of 200 mL of distilled water and then filtration. Afterward, the filtrate was titrated with 0.5 N Fe_2_SO_4_.7H_2_O until reaching a dark brown color, indicating the end point. The hydrometer method was used to determine soil texture^52^. Air dried soil sample (50 g) was taken in a dispersion cup. Distilled water and 10 mL of 1 N Na_2_CO_3_ were added to it and through dispersing machine, it was shaken for 5–10 min. For silt, hydrometer reading was recorded after 40 s, while for clay, the reading was taken after two hours. The quantity of sand was determined by differences.

Inorganic soil nitrogen was determined by the method of Bremner and Mulvaney^53^ that uses a 1 M KCl extraction followed by Kjeldhal distillation in the presence of MgO + devarday’s alloy and then titration to determine total N.

Total nitrogen in plant and soil samples was done by the Kjeldhal method as described by Bremner and Mulvaney^53^. In brief finely ground soil or plant sample samples were digested at an elevated temperature with H_2_SO_4_ followed by distillation and titration to determine N content.

### Statistical analysis

ANOVA was performed to detect treatment effects for the various soil and plant parameters that were measured. Means separation analysis was done using a modified Tukey LSD method. Statistical analysis was performed using Minitab (version 12)^54^.

### Plant material collection and use permission

No permission is required for plant material as it was purchased from certified dealer of local area.

### Ethics approval and consent to participate

We all declare that manuscripts reporting studies do not involve any human participants, human data, or human tissue. So, it is not applicable.

### Complies with international, national and/or institutional guidelines

Experimental research and field studies on plants (either cultivated or wild), comply with relevant institutional, national, and international guidelines and legislation.
